# Therapy With Intravenous Methylprednisolone Pulses Is Associated With Loss of Bone Microarchitecture in Trabecular Bone Score -Assessment Among Patients With Moderate-to-Severe Graves’ Orbitopathy: A Pilot Study

**DOI:** 10.3389/fendo.2022.893600

**Published:** 2022-07-14

**Authors:** Joanna Rymuza, Katarzyna Pelewicz, Jerzy Przedlacki, Piotr Miśkiewicz

**Affiliations:** ^1^ Department of Internal Medicine and Endocrinology, Medical University of Warsaw, Warsaw, Poland; ^2^ Department of Nephrology, Dialysis and Internal Medicine, Medical University of Warsaw, Warsaw, Poland

**Keywords:** graves’ orbitopathy, graves’ ophthalmopathy, trabecular bone score, EUGOGO, methylprednisolone

## Abstract

**Background:**

Therapy with intravenous glucocorticoids (GCs) is associated with various side effects, however, the impact on bone remains elusive. Trabecular bone score (TBS) is a diagnostic tool providing information on bone microarchitecture based on images obtained from dual-energy X-ray absorptiometry. We investigated the influence of the intravenous methylprednisolone (IVMP) pulse administration on TBS in patients with moderate-to-severe Graves’ orbitopathy (GO).

**Methods:**

Fifteen patients with GO were treated with 12 IVMP pulses (6x0.5g, 6x0.25 g on a weekly schedule). They received supplementation with 2000 IU of vitamin D and 1.0 g of calcium throughout the study period. TBS was assessed at baseline and after last IVMP pulse. To determine the difference between values at baseline and after treatment the least significant change (LSC) methodology was used. We compared pre- and posttreatment mean TBS values.

**Results:**

We found a significant decrease of TBS in 5 out of 15 (33%) patients. Mean TBS value decreased becoming 2.4% lower than at baseline (p<0.05).

**Conclusions:**

IVMP pulse therapy exerts negative effect on bone microarchitecture in TBS assessment. The analysis of the clinical risk factors for osteoporosis and the evaluation of bone mineral density and TBS should be considered before initiating IVMP therapy.

## Introduction

Glucocorticoids (GCs) are highly effective and widely used for treatment of various autoimmune diseases. Therapy with GCs is associated with multiple side effects (1–5), including the negative impact on bone, leading to secondary osteoporosis and an increased risk of fractures (6, 7).

GCs affect bone in several ways: they decrease calcium absorption in the gut (8), reduce calcium reabsorption in the kidney (9), decrease production of sex hormones (10), cause proximal muscle weakness (11). However, the main negative mechanism involves the increase of the osteoclast lifespan, stimulation of the osteocyte and the osteoblast apoptosis, and the decrease of the osteoblastogenesis. These processes lead to enhanced bone resorption, decreased bone formation, and finally reduction of bone quality and structure (12, 13).

GCs have probably a greater impact on bone microarchitecture rather than on bone mass (14–16). Among patients treated with oral GCs individual fracture risk is increased independently of bone mineral density (BMD) as the fractures often occur with non-osteoporotic T-score values (10, 17). The effects of GCs on bone quality are not fully expressed by BMD measurement (14, 16, 18). Therefore, to identify GCs-treated patients with high risk of fracture, the analysis of other factors contributing to bone strength and resistance to fracture is needed.

The trabecular bone score (TBS) is a non-invasive technique that performs novel gray-level texture measurements on lumbar spine DXA images, and thereby enables estimating trabecular microstructure and assessment of bone quality (19). Low TBS values indicate weak bone, prone to fractures with fewer poorly connected trabeculae, whereas elevated TBS values reflect denser bone with stronger bone microarchitecture. As shown in previous studies, TBS might be a good indicator of bone health in patients treated with GCs, as it seems to be more sensitive than BMD in detecting the GCs-induced fractures (14, 20–22).

Although the negative impact of oral GCs on bone microarchitecture was demonstrated in several studies (18, 20–22), the influence of intravenous GCs remains elusive. A few studies suggest no negative impact of intravenous methylprednisolone (IVMP) on BMD (23–26). Others demonstrate loss of BMD (27, 28). There is only one pilot study reporting no change of TBS after IVMP pulse therapy (29).

GCs administered intravenously are commonly used in a variety of autoimmune diseases. IVMP therapy in weekly infusions is still the first-line treatment according to the latest European Group on Graves’ Orbitopathy (EUGOGO) recommendations in patients with moderate-to-severe and active Graves’ orbitopathy (GO) (30–32). Throughout the therapy bone protection is recommended (32). The aim of our study was to investigate early changes of TBS after IVMP therapy with cumulative dose of 4.5 g in euthyroid patients with active, moderate-to-severe GO.

## Materials and Methods

### Patients

Consecutive patients with active, moderate-to-severe GO were enrolled to participate in the study between 2018 to 2021. GO was diagnosed and treated according to the EUGOGO recommendations (33). The study was conducted at the Department of Internal Medicine and Endocrinology, Medical University of Warsaw. Exclusion criteria were: age < 20 years; BMI < 17 kg/m2 or > 37 kg/m2; treatment with oral or intravenous GCs within the last 6 months; any other treatment known to significantly alter bone metabolism (i.e. bisphosphonates or other drugs with anti-fracture effects, heparin, vitamin-K antagonists, proton pump inhibitors, selective serotonin reuptake inhibitors, benzodiazepines, antiepileptic, antipsychotic drugs); clinical diagnosis of osteoporosis based on the presence of low-energy fractures, or BMD measurements (DXA T score below -2.5 SD), as defined by the World Health Organization (WHO) (34). We included to the study 15 patients: 14 patients diagnosed with Graves’ disease and 1 patient with Hashimoto thyroiditis. Among patients with Graves’ disease, 11 patients were treated with antithyroid drugs (alone or according to a “block and replace” schedule) and 3 individuals who were at least 6 months after the last radical treatment (thyroidectomy and/or radioiodine therapy) received levothyroxine. One patient had Hashimoto thyroiditis treated with levothyroxine. All patients remained clinically euthyroid, with free triiodothyronine (fT3) and free thyroxine (fT4) levels within the reference range at baseline, in the last month prior to the study as well as throughout the time of the IVMP therapy. All patients were treated with IVMP pulses in a 12-week protocol (six infusions of 0.5 g, followed by six infusions of 0.25 g; cumulative dose 4.5 g) according to the current EUGOGO recommendations (33). Supplementation with 2000 IU of vitamin D and 1.0 g of calcium daily was routinely initiated in all patients at the beginning of IVMP therapy and continued throughout the study. The 25-hydroxyvitamin D [25(OH)D] concentrations below 20 ng/mL were described as deficient, concentrations of 20-30 ng/mL as suboptimal, and concentrations higher than 30 ng/mL as optimal vitamin D status, based on the guidelines for vitamin D supplementation and treatment of deficits approved in Central Europe (35). The baseline characteristics of the study group are presented in **Table 1**. All procedures were performed in accordance with the 1964 Helsinki declaration. Informed and written consent was obtained from all individual participants included in the study. The study was approved by the Local Bioethics Committee (KB/197/2018).

**Table 1:** Baseline characteristics of patients (n = 15).

Characteristic	Number of patients (%) or mean ± SD (range)
Age, years	53.6 ± 10.6 (40 ÷74)
Male/female	2/13 (13%/87%)
Menopause (in women)	6 (46%)
Years after menopause (in women)	15.3 ± 15.3 (5.0 ÷ 30.0)
Body mass index (kg/m2)	28.0 ± 6.0 (20.3 ÷ 37.0)
Current smokers	6 (40%)
**Thyroid disease**	
Duration of thyroid disease (years)	3.6 ± 6.9 (0.3 ÷ 24.0)
Graves’ disease treated for hyperthyroidism	11 (73%)
Graves’ disease after radical treatment on levothyroxine	3 (20%)
Hashimoto thyroiditis on levothyroxine	1 (7%)
Duration of therapy with antithyroid drugs (months)^a^	8.6 ± 4.4 (4.0 ÷ 18.0)
TSH (reference range: 0.27–4.2 μIU/mL)	1.8 ± 1.7 (0.005 ÷ 5.1)
fT4 (reference range:12.0–22.0 pmol/L)	15.3 ± 2.7 (12.0 ÷ 21.1)
fT3 (reference range: 3.1–6.8 pmol/L)	4.3 ± 0.9 (3.1 ÷ 6.2)
TRAb (reference range: <1.8 IU/l)	13.5 ± 12.5 (1.7÷ 40.0)
25(OH)D (ng/mL)	32.3 ± 12.3 (13.8 ÷ 57.0)
DXA lumbar spine: BMD (g/cm2)	1.05 ± 0.1 (0.89 ÷ 1.25)
DXA lumbar spine: T-score (SD)	-0.01 ± 1.1 (-1.6 ÷ 1.8)
DXA lumbar spine: Z-score (SD)	0.99 ± 1.6 (-1.2 ÷ 4.1)
DXA femoral neck: BMD (g/cm2)	0.83 ± 0.1 (0.61 ÷ 0.93)
DXA femoral neck: T-score (SD)	-0.23 ± 0.7 (-2.1 ÷ 0.6)
DXA femoral neck: Z-score (SD)	0.61 ± 0.8 (-0.7 ÷ 2.5)
Densitometric osteopenia	4 (27%)
Lumbar spine: osteopenia	3 (20%)
Femoral neck: osteopenia	2 (13%)
TBS for vertebrae L1-L4	1.31 ± 0.2 (0.92 ÷ 1.50)
Partially disturbed microarchitecture	2 (13%)
Degraded microarchitecture	3 (20%)

### BMD and TBS Evaluation

Areal BMD (grams per cm2) of the lumbar spine (L1–L4) and the femoral neck were assessed using dual-energy X-ray absorptiometry (DXA) at baseline (within 2 weeks before IVMP therapy) and within one month after the 12^th^ IVMP pulse. DXA scans were performed by one technician using the Hologic Discovery A Densitometer. BMD measurements were calculated, and Z-scores and T-scores were subsequently analyzed by the same physician. Osteoporosis and osteopenia were diagnosed in individuals with a T-score of the lumbar spine and/or the femoral neck ≤−2.5 standard deviation (SD) and between <−1.0 and >−2.5 SD, respectively (34).

TBS was calculated using iNsight^®^ Software (version 3.0, Med-Imaps, Pessac, France) on the DXA lumbar spine (L1-L4) images. The TBS absolute values for the sum of vertebrae L1-L4 were reported. The absolute TBS values <1.230 were considered as the degraded microarchitecture, TBS values between ≥ 1.23 and <1.31 indicated partially disturbed bone microarchitecture, whereas TBS values ≥ 1.31 were assessed as normal (36).

The least significant change (LSC) methodology was used to evaluate the differences in BMD expressed in g/cm^2^ and TBS values before and after the IVMP treatment. LSC values for BMD and TBS were calculated for the DXA device in the Medical University of Warsaw’s densitometry unit and were estimated to be 3% for the lumbar spine BMD, 5.4% for the femoral neck BMD and 4.6% for TBS. An increase or decrease of BMD or TBS equal to or exceeding the LSC was considered significant.

### Laboratory Evaluation

Thyroid-stimulating hormone (TSH), fT3, fT4, thyrotropin receptor antibodies (TRAb) and 25(OH)D levels were assessed at baseline and after the last IVMP pulse. TSH, fT3, fT4, TRAb and 25(OH)D were examined using an electrochemiluminescence immunoassay on Cobas 8000 Analyzer (Roche Diagnostics, Mannheim, Germany). The reference ranges for TSH, fT3, fT4 and TRAb were: 0.27–4.2 μIU/mL, 3.1–6.8 pmol/L, 12.0–22.0 pmol/L and <1.8 IU/mL, respectively.

### Statistical Analysis

All analyses were performed using SPSS statistical software version 22.0 (IBM SPPS Statistics, New York, US). Continuous variables are expressed as means ± standard deviation (SD), while categorical variables are expressed as numbers (n) and percentages (%). The Shapiro-Wilk test was used to confirm or reject the normal distribution of each continuous variable. Comparisons between continuous data were performed using paired t-test (for parameters with normal distribution) or Wilcoxon rank sum test (for parameters with distribution deviations). Categorical data were analyzed using Fisher exact test. Comparisons between continuous data were performed with the Mann – Whitney U test. Pearson correlation test was performed to investigate correlations. Statistical significance was established for results with p<0.05.

## Results

### Baseline Data

At baseline, 3 out of 15 patients (20%) had degraded, and 2 out of 15 patients (13%) had partially disturbed microarchitecture. We found osteopenia in 4 out of 15 patients (27%): in 2 patients only in the lumbar spine BMD, in 1 patient only in the femoral neck BMD and in another patient in both measurement sites. The baseline TBS values correlated negatively with BMI (r=-0.72, p=0.003). We observed vitamin D deficiency in 2 patients.

### Effect of IVMP Therapy on TBS and BMD

**Figure 1:**
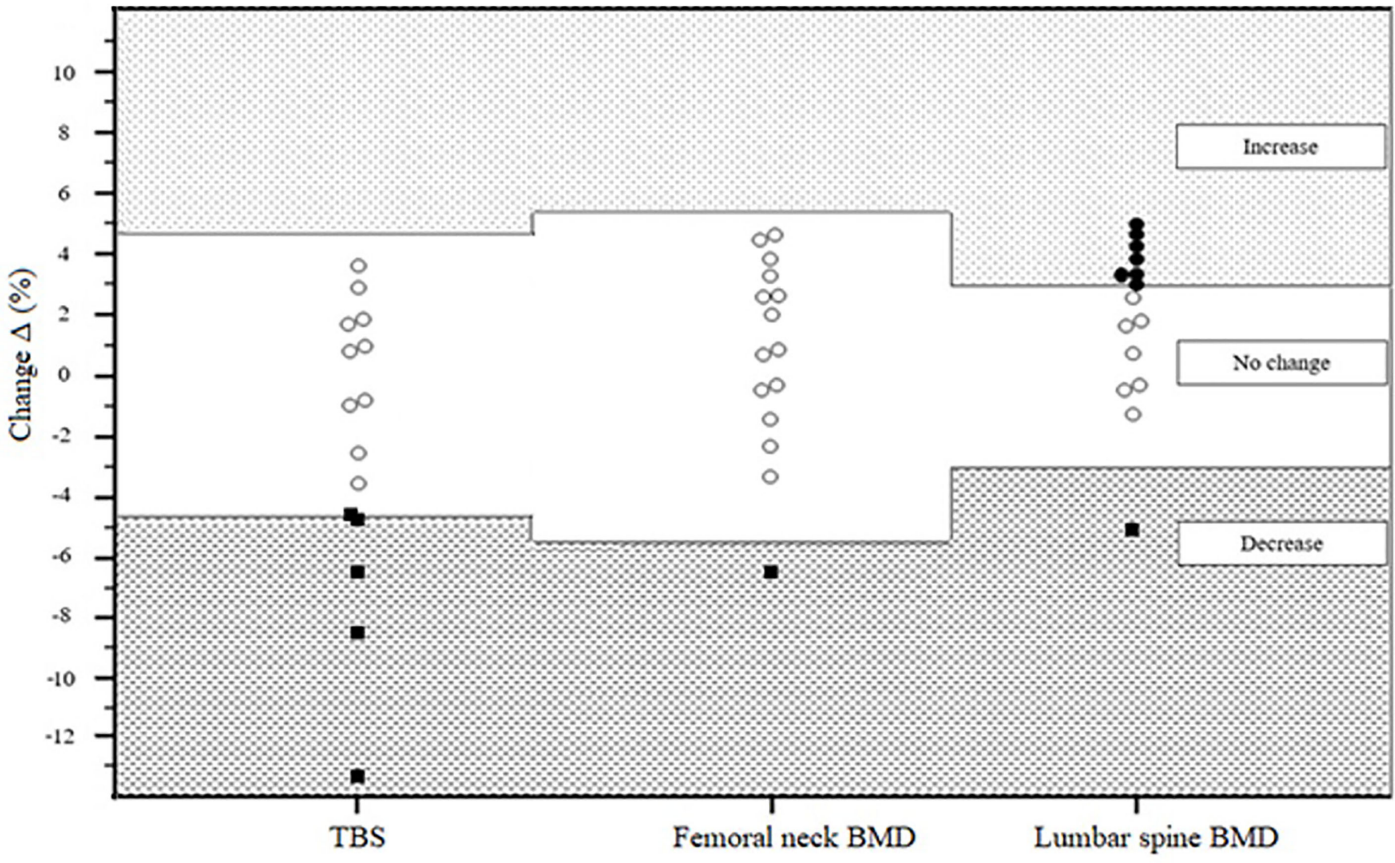
Percentage of TBS and BMD changes according to the LSC criteria in 15 patients after IVMP therapy. TBS trabecular bone score BMD bone mineral density LSC least significant change IVMP intravenous methylprednisolone. Bullets and squares represent individual percentage of BMD and TBS changes (black squares represent a decrease in TBS or BMD – equal to or exceeding LSC calculated to be 4.6% change for TBS, 5.4% change for femoral neck and 3% change for lumbar spine; black bullets represent an increase in BMD equal to or exceeding LSC; white bullets represent no change in TBS or BMD). BMD, bone mineral density; IVMP, intravenous methylprednisolone therapy; LSC, least significant change; TBS, trabecular bone score.

According to the LSC criteria, we found the following changes in TBS and BMD after 12 weeks of IVMP therapy (**Figure 1**):

- decrease in TBS in 5 out of 15 patients (33%)
- decrease of BMD in 2 out of 15 patients (13%; 1 in the lumbar spine BMD, 1 in the femoral neck BMD)
- increase of BMD in 7 out of 15 patients (47%; all in the lumbar spine BMD)
- no increase of the femoral neck BMD
- no increase of TBS.

**Figure 2:**
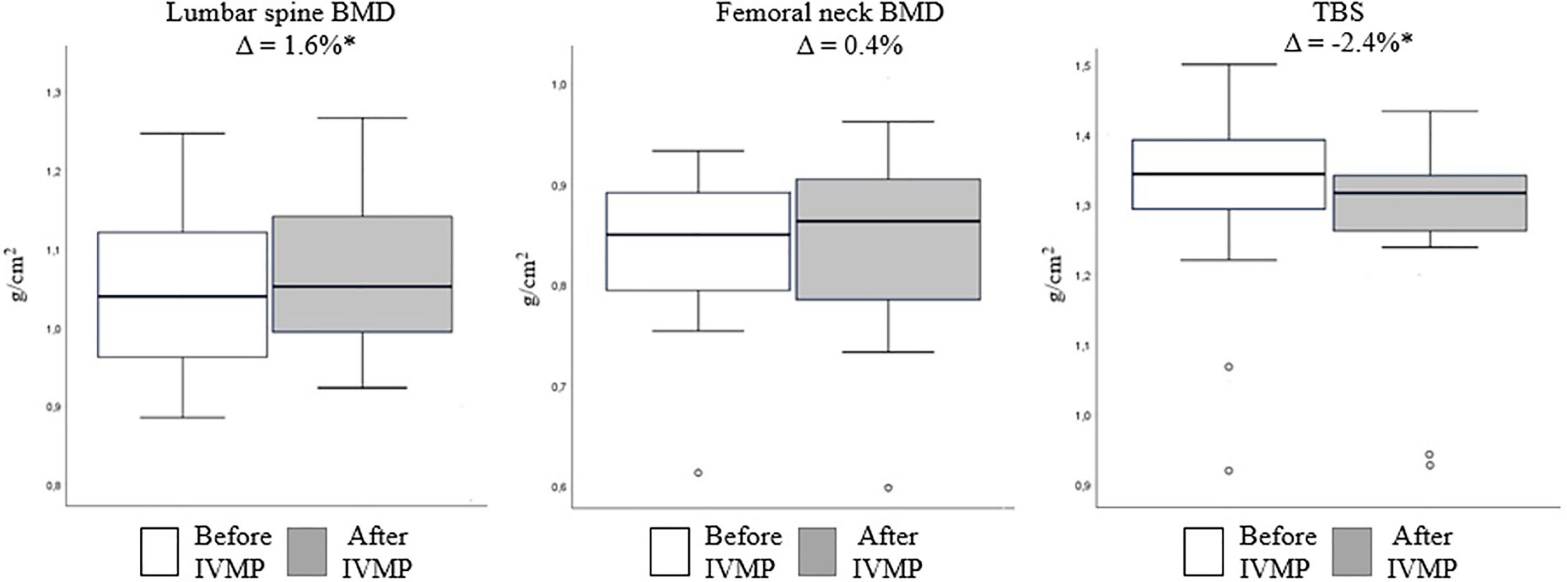
Mean bone mineral density and trabecular bone score values before (white) and after (gray) intravenous methylprednisolone (IVMP) therapy. Values shown above are Δ, calculated as 100 x (after IVMP value – before IVMP value)/before IVMP value. Data are expressed as mean ± SD. *p<0.05 vs. baseline. BMD, bone mineral density; TBS, trabecular bone score; IVMP, intravenous methylprednisolone therapy; SD, standard deviation.

Mean TBS value decreased becoming 2.4% lower than at baseline (p=0.04). Mean lumbar spine BMD increased becoming 1.6% higher after IVMP therapy than at baseline (p=0.047). There was no significant change in mean post-treatment femoral neck BMD as compared to the baseline. (**Figure 2**, **Table 2**).

**Table 2:** Comparison of bone mineral density and trabecular bone score between baseline and after intravenous methylprednisolone therapy (n = 15).

Variable	Before IVMP	After IVMP	P
Lumbar spine BMD (g/cm^2^)	1.05 ± 0.11	1.07 ± 0.11	0.047
Femoral neck BMD (g/cm^2^)	0.83 ± 0.08	0.84 ± 0.10	0.43
TBS	1.31 ± 0.15	1.28 ± 0.16	0.04

Continuous variables are presented as means ± SD.

BMD, bone mineral density; IVMP, intravenous methylprednisolone; SD, standard deviation; TBS, trabecular bone score.

The correlations between the baseline TBS values and the changes in TBS with selected parameters are presented in **Table 3**.

**Table 3:** Correlations between the baseline trabecular bone score (TBS) and change in TBS with selected parameters.

Parameter	Baseline TBS	Change in TBS
Age	r=0.06, p= 0.84	r=-0.18, p=0.52
Baseline BMI	r=-0.72, p=0.003	r=-0.10, p=0.71
Baseline TRAb	r=0.18, p= 0.52	r=-0.32, p=0.25
Baseline TSH	r=-0.01, p= 0.97	r=0.01, p=0.99
Baseline 25(OH)D	r=0.35, p=0.20	r=-0.03, p=0.93
Baseline lumbar spine BMD	r=0.42, p=0.12	r=0.12, p=0.67
Change in lumbar spine BMD	r=0.23, p=0.41	r=0.26, p=0.35
Baseline femoral neck BMD	r=-0.27, p=0.33	r=0.04, p=0.90
Change in femoral neck BMD	r=-0.26, p=0.34	r=0.02, p=0.95
Duration of therapy with antithyroid drugs^a^	r=-0.15, p=0.65	r=0.31, p=0.31

a Analysis performed in 11 patients treated with antithyroid drugs throughout the study.

BMD, bone mineral density; BMI, body mass index; IVMP, intravenous methylprednisolone; TBS, trabecular bone score; TRAb, TSH receptor antibodies; TSH, thyroid-stimulating hormone.

There were no significant differences between the groups with decreased TBS (drop in TBS equal to or exceeding the LSC) vs. no change in TBS after IVMP treatment as far as the selected characteristics were considered (**Table 4**).

**Table 4:** Comparison of selected baseline characteristics of patients with and without decrease of trabecular bone score (TBS) (decrease in TBS equal to or exceeding the least significant change) after intravenous methylprednisolone therapy.

Characteristic	Decrease of TBS 5/15 (33%)	No change of TBS 10/15 (66%)	P
Age (years), mean ± SD	59.0 ± 13.8	50.9 ± 8.1	0.33^b^
Women, n (%)	5 (100%)	8 (80%)	0.52^a^
Women after menopause, n (%)	3 (60%)	3 (30%)	0.59^a^
BMI (kg/m^2^),	27.8 ± 6.3	27.4 ± 4.8	0.90^b^
Smokers, n (%)	2 (40%)	4 (40%)	1.00^a^
TSH (µIU/mL), mean ± SD	1.5 ± 2.0	1.9 ± 1.6	0.76^b^
TRAb (IU/L), mean ± SD	20.0 ± 17.4	10.3 ± 8.6	0.27^b^
25(OH)D (ng/mL), mean ± SD	27.5 ± 7.0	34.7 ± 14.0	0.46^b^
Osteopenia (T score -1.0 to >-2.5), n (%)	2 (40%)	2 (10%)	0.56 ^a^
Degraded or partially disturbed microarchitecture (TBS <1.31), n (%)	1 (20%)	4 (40%)	0.60^a^
Lumbar spine BMD (g/cm^2^), mean ± SD	1.040 ± 0.1	1.061 ± 0.1	0.81^b^

a Chi-squared Test;

b Mann – Whitney U test.

BMD, bone mineral density; BMI, body mass index; TBS, trabecular bone score; TSH, thyroid-stimulating hormone; TRAb, TSH receptor antibodies; SD, standard deviation; 25(OH)D, 25-hydroxyvitamin D.

In **Table 1** (of the **Supplementary Material**) we have included the results of BMD and TBS of our study group before and after treatment with IVMP pulses.

A decrease of TBS equal to or exceeding LSC value occurred in 2 out of 2 women and in none out of 2 men with osteopenia before IVMP therapy. In one woman with degraded TBS a decrease exceeding LSC value occurred. There was no correlation between the changes in TBS and the baseline TBS values (r =-0.03, p=0.91). However, no correlation was found between the changes of TBS and the baseline lumbar spine BMD, or the femoral neck BMD. Details are presented in **Table 3**.

There was no significant difference in serum 25(OH)D concentration. No correlation was found between changes in TBS and baseline vitamin D status.

## Discussion

The presented study revealed a decrease of the mean TBS value in patients with GO treated with IVMP. The reduction of TBS value equal to or exceeding the LSC occurred in 33% of the patients. In contrast, we observed the overall increase of the mean BMD in the lumbar spine as well as the increase in the lumbar spine BMD exceeding the LSC value in almost half of the study group.

This is the first study showing the negative effect of the intravenous GCs on bone microarchitecture. The current results differ from research performed by Censi et al. involving GO patients treated with IVMP pulse therapy, in which no change in neither of TBS nor BMD was found (29). The possible explanations for the divergent results include different cumulative doses of IVMP (4.5 g of IVMP in the current research vs. 1.5-5.25 g of IVMP in Censi et al. study), different timing of follow-up and no analysis according to the LSC criteria in Censi et al. research. The results of our research stay in agreement with recent studies involving patients treated with oral GCs that demonstrate the decline of TBS while BMD remains unchanged (20–22). The trabecular bone microarchitecture seems to be more affected than BMD not only in patients treated with oral but with intravenous GCs as well. The findings of our study bring evidence that the adverse effects of IVMP on bone are not fully captured with BMD evaluation and that TBS adds value to the BMD assessment.

Based on previous studies, the decrease in bone quality plays a significant role in the rapid increase in fracture risk occurring in GCs-treated patients. Fractures may be better predicted when TBS is used in addition to BMD (37–40). It highlights the clinical need for methods that can identify GCs-treated individuals particularly vulnerable to steroid-induced deterioration of bone quality, as those are at increased risk of fractures. The results of current study indicate that the decrease in TBS despite the increase of BMD might be an indicator that the intravenous GCs are not so safe in terms of bone safety. Further studies with a larger study group should include vertebral and non-vertebral fracture assessment during therapy as well as in the follow-up period.

Some of the clinical risk factors need to be taken into consideration when assessing the influence of GCs on bone microarchitecture. We found a negative correlation of baseline TBS with BMI. This stays in agreement with other reports suggesting that lower TBS values are present more frequently in patients with higher BMI (41). However, we noticed no correlation between change in TBS and BMI. We found no correlations between baseline TBS, or change in TBS and baseline levels of TSH, fT4, fT3, TRAb or duration of treatment with antithyroid drugs. There were no differences in baseline TSH levels between subjects with a decrease versus those lacking any change in TBS.

Another issue that should be considered is that Graves’ disease itself has been demonstrated to have a strong correlation with decreased TBS due to the hyperthyroidism that increases bone resorption (42). Bone loss is enhanced because of the thyrotoxicosis as well as the excessive release of inflammatory cytokines (43). In the present study, all the patients had documented fT4 and fT3 within the reference range for at least one month before the first IVMP infusion as well as during the whole therapy. During the observation time the majority of patients were treated with antithyroid therapy, which had been initiated without delay in the past. Previous studies concerning the reversibility of bone loss after the initiation of antithyroid therapy have shown that bone quantity measured by BMD as well as bone quality measured using TBS improve simultaneously after thyroid function normalization (44, 45). The increase in BMD after IVMP therapy observed in current as well as in our previous study (23) were greater for sites rich in trabecular bone (lumbar spine) than with cortical bone (femoral neck), as trabecular bone is more metabolically active, and changes occur earlier than in cortical bone (46). Taking everything into consideration, we cannot exclude that the BMD changes during the therapy with intravenous GCs were secondary to the ongoing restoration of BMD after the stabilization of thyroid function as well as the reduction of the inflammatory state. However, the decrease of TBS occurring simultaneously with the increase of lumbar spine BMD indicates that in the course of the IVMP therapy the restored bone density might have been unevenly distributed within the trabecular bone. Further study and analysis are needed in order to confirm our observations as well as to identify patients particularly vulnerable to bone quality deterioration during IVMP therapy.

There are studies indicating that the supplementation with vitamin D might be effective in preventing of bone loss in GCs-treated patients (47). In our study the mean vitamin D status at baseline was optimal, the supplementation with 1.0 g of calcium and 2000 IU of vitamin D was continued through the whole study period. We did not observe any correlations between changes in TBS and baseline vitamin D status. There was also no difference between groups with and without decrease of TBS equal to or exceeding LSC as far as baseline 25(OH)D concentration was taken into account. It is noteworthy that despite the optimal vitamin D status at baseline and the sufficient vitamin D prophylaxis, the decrease in TBS could not be prevented.

The biggest limitation of our study is a small sample size including men, pre- and postmenopausal women, different age groups and the reassessment of TBS after a relatively short follow-up period. However, the strength of our study is the therapy of all subjects according to the same protocol. We consider this research as a pilot study that allows us to design larger prospective research with longer follow-up period. More studies with additional measurements of TBS and fracture assessment are needed to determine whether TBS improves with time and if the fractures occur in patients after IVMP therapy. Patients with GO sometimes require the second-line treatment including, among others, the second course of IVMP with a higher cumulative dose (7.5 g) or oral GCs, which may potentially further exacerbate bone microarchitecture. Finally, TBS is an indirect index of bone microstructure state, and the accuracy of TBS measurements might be affected by body composition (48). However, each patient served as his or her own control.

It is noteworthy that according to the latest EUGOGO recommendations (32), in the most severe forms of moderate-to-severe and active GO, a higher cumulative dose of IVMP (7.5 g) is recommended as an alternative first-line treatment. Further studies aimed to assess bone microarchitecture in patients treated with different cumulative doses of IVMP (4.5 g vs. 7.5 g) are needed. The assessment of the impact of IVMP on bone markers would have also a great value as the mechanisms through which IVMP exerts its effects on bone remain not fully recognized.

In conclusion, our study revealed that IVMP pulse therapy with cumulative dose of 4.5 g is associated with loss of bone microarchitecture in TBS assessment with no negative effect on BMD among patients with GO.

## Data Availability Statement

The original contributions presented in the study are included in the article/**Supplementary Material**. Further inquiries can be directed to the corresponding author.

## Ethics Statement

The studies involving human participants were reviewed and approved by Local Bioethical Committee of the Medical University of Warsaw. The patients/participants provided their written informed consent to participate in this study.

## Author Contributions

JR and PM were responsible for the concept and design of the study. JR and KP collected patient data and prepared the data for analysis. JR, KP, JP, and PM analyzed clinical data. JR was responsible for statistical analysis, tables, and figures. All authors drafted the manuscript. PM supervised all aspects of the study, critically revised the manuscript and approved the final manuscript as submitted. All authors contributed to the article and approved the submitted version.

## Conflict of Interest

The authors declare that the research was conducted in the absence of any commercial or financial relationships that could be construed as a potential conflict of interest.

## Publisher’s Note

All claims expressed in this article are solely those of the authors and do not necessarily represent those of their affiliated organizations, or those of the publisher, the editors and the reviewers. Any product that may be evaluated in this article, or claim that may be made by its manufacturer, is not guaranteed or endorsed by the publisher.
