# Supplementary material for: Therapy With Intravenous Methylprednisolone Pulses Is Associated With Loss of Bone Microarchitecture in Trabecular Bone Score -Assessment Among Patients With Moderate-to-Severe Graves’ Orbitopathy: A Pilot Study
Source: Front Endocrinol (Lausanne). 2022 Jul 14;13:893600. doi: 10.3389/fendo.2022.893600 (PMC9331277; doi:10.3389/fendo.2022.893600)
Supplement: Supplementary file 1 [file Table_1.docx]

**Supplementary Table 1.** Results of LS BMD, FN BMD and TBS measurements before and after IVMP therapy

in the study group.

| **No.** | **Sex** | **Age** | **LS BMD** | | | **FN BMD** | | | **TBS** | | |
| --- | --- | --- | --- | --- | --- | --- | --- | --- | --- | --- | --- |
|  |  |  | Before IVMP | After IVMP | Δ (%) | Before IVMP | After IVMP | Δ (%) | Before IVMP | After IVMP | Δ (%) |
| 1 | M | 40 | 0.952 | 0.997 | **4.7** | 0.772 | 0.775 | 0.4 | 1.311 | 1.331 | 1.5 |
| 2 | F | 63 | 1.008 | 1.000 | -0.8 | 0.817 | 0.804 | -1.6 | 1.221 | 1.259 | 3.1 |
| 3 | F | 71 | 0.972 | 1.017 | **4.6** | 0.613 | 0.598 | -2.4 | 1.352 | 1.266 | **-6.4** |
| 4 | F | 45 | 1.148 | 1.198 | **4.4** | 0.888 | 0.909 | 2.4 | 1.303 | 1.321 | 1.4 |
| 5 | F | 74 | 1.241 | 1.267 | 2.1 | 0.896 | 0.897 | 0.1 | 1.501 | 1.432 | **-4.6** |
| 6 | F | 54 | 1.203 | 1.183 | -1.7 | 0.833 | 0.863 | 3.6 | 1.467 | 1.480 | 0.9 |
| 7 | F | 47 | 1.094 | 1.101 | 0.6 | 0.850 | 0.795 | **-6.5** | 1.343 | 1.307 | -2.7 |
| 8 | F | 40 | 1.029 | 1.060 | **3.0** | 0.882 | 0.900 | 2.0 | 1.403 | 1.353 | -3.6 |
| 9 | F | 54 | 0.950 | 0.979 | **3.1** | 0.827 | 0.826 | -0.1 | 1.438 | 1.420 | -1.3 |
| 10 | M | 51 | 0.918 | 0.947 | **3.2** | 0.904 | 0.941 | 4.1 | 0.919 | 0.941 | 2.4 |
| 11 | F | 63 | 1.247 | 1.268 | 1.7 | 0.758 | 0.733 | -3.3 | 1.344 | 1.330 | -1.0 |
| 12 | F | 52 | 1.065 | 1.079 | 1.3 | 0.895 | 0.933 | 4.2 | 1.383 | 1.317 | **-4.8** |
| 13 | F | 44 | 1.060 | 1.051 | -0.8 | 0.880 | 0.901 | 2.4 | 1.284 | 1.291 | 0.5 |
| 14 | F | 46 | 0.885 | 0.919 | **3.8** | 0.754 | 0.751 | -0.4 | 1.358 | 1.239 | **-8.8** |
| 15 | F | 60 | 1.039 | 0.986 | **-5.1** | 0.933 | 0.962 | 3.1 | 1.068 | 0.926 | **-13.3** |
| Δ – percentage change between FN BMD, LS BMD and TBS before and after IVMP therapy  Δ was calculated with the following equation:  Δ = (BMD or TBS after IVMP – BMD or TBS before IVMP) : BMD or TBS before IVMP (%)  Δ equal to or exceeding LSC is bolded  Abbreviations: BMD, bone mineral density; F, female; FN BMD, femoral neck BMD; IVMP, intravenous methylprednisolone; LS BMD, lumbar spine BMD; M, male; No., number | | | | | | | | | | | |
